# A meta-ethnography of shared decision-making in mental health care from the perspective of staff and service users

**DOI:** 10.1186/s12913-024-11540-9

**Published:** 2024-09-27

**Authors:** Claire Cartwright, Beth Greenhill, Alys Wyn Griffiths, John Harrison

**Affiliations:** 1 Department of Primary Care and Mental Health, University of Liverpool, Liverpool, UK; 2 Department of Neuroscience, University of Sheffield, Sheffield, UK; 3 Eleanor Rathbone Building, The University of Liverpool, Bedford Street South, Liverpool, L69 7ZA UK

**Keywords:** Shared decision making, Mental health, Staff, Service users

## Abstract

**Background:**

Human rights, recovery, and value-based approaches are integral to strategic changes and development in mental health care. Successfully integrating such person-centred values in mental health services requires a paradigm shift from traditional biomedical models of care to a more human rights-based approach. An important aspect of this is shared decision making (SDM) between mental health staff and service users. Whilst it is widely acknowledged SDM leads to improved outcomes, there are barriers and challenges to implementing this approach effectively in clinical practice.

**Objectives:**

This systematic review aimed to assess existing empirical research exploring mental health service users and/or staff’s attitudes towards and experiences of SDM in adult mental health care settings.

**Methods:**

The review and protocol were registered on PROSPERO (CRD42023369472). Systematic searches were run on four databases. Search terms pertained to studies reporting on mental health staff or service users’ experiences of SDM in adult mental health care. Initial searches yielded 721 results. Included studies were analysed using a meta-ethnographic approach.

**Results:**

Thirteen articles were included. Data were synthesised using meta ethnographic synthesis, which produced four higher order themes with related subthemes; the role of service user ownership, the influence of fluctuating capacity, the importance of therapeutic alliance and changing clinicians’ behaviours and attitudes.

**Implications:**

Both staff and service users found SDM to be an important factor in delivering high quality, effective mental health care. Despite this, participants had very little experience of implementing SDM in practice due to several personal, professional, and organisational challenges. This suggests that differences exist between what services strive towards achieving, and the experience of those implementing this in practice. These findings suggest that further research needs to be conducted to fully understand the barriers of implementing SDM in mental health services with training delivered to staff and service users about SDM.

Health and social care professionals tend to hold most responsibility for people in their care when there are concerns around risk, safety, and the person’s capacity to keep themselves or others safe and well [1]. As a result, people who are patients or service users in mental health services particularly report having very little to no choice or control in their day-to-day care [2]. Service users understandably report having difficult emotional responses to their limited involvement in their care, including a lack of understanding for the rationale of certain decisions [3]. Conversely, caring for individuals in mental health settings presents challenges for healthcare professionals, including difficulties building a therapeutic rapport with a person whilst enforcing boundaries or restrictions. This is particularly prominent within inpatient settings [4]. These issues together mean that difficulties can arise in the relationship between service users and healthcare professionals.

Inpatient care must ensure the safety and well-being of people experiencing mental health difficulties which place either themselves or the public at an increased risk of harm [4, 5]. Most people admitted to inpatient mental health settings are there involuntarily, i.e. against their will or decision [6]. In this situation, the ability to actively contribute to decisions related to their care is often denied to service users [7]. Therefore, their right to confidentiality, right to give informed consent and to express autonomy are all removed. Within the United Kingdom, decisions to detain people under the Mental Health and Act (1983) [8] involve a series of complex ethical issues and balances to protect the individual’s human rights whilst also keeping the person safe and protecting the public. The responsible clinician’s right to enforce medical treatment to ensure the individual’s safety is often referred to as a ‘duty of care’ to the service user. However, under certain circumstances, particularly where coercive interventions have been used for example, implicit or explicit pressure to accept certain treatments including forced practices such as involuntary admission, control or restraint may compromise the service user’s dignity [3, 9].

When service users are detained under the Mental Health Act (MHA) their levels of distress can be demonstrated through physical behaviour [10]can lead to nursing staff using interventions such as control and restraint, seclusion or rapid tranquillisation. The Care Quality Commission has a statutory duty to safeguard detained service users and guidelines on techniques to be used in the management of violence and aggression which state that such coercive interventions should be a last resort. Although mental health legislation differs across the world in terms of legality surrounding involuntary admissions, there are commonalities in balancing duties of care, protection and human rights [11].

Shared decision making (SDM) places an increased emphasis on a partnership between service users and staff providing service users with enhanced control and choice as part of a more personalised approach to care [12]. Definitions vary, but broadly shared decision making involves a sense of warmth and openness, emotional bond, and shared expectations of the goals and tasks of therapy [13]. A key element, and perhaps linked to that of shared expectations, is mutual collaboration [14]. There is overlap both in the language and practical application of therapeutic alliance and shared decision making. As such, SDM aims to represent a balancing of power within the mental health system, valuing the patient or service user’s experiential and healthcare professional’s medical expertise more equally [15]. Traditionally, mental health services have employed paternalistic approaches to assessing and managing service users risk status with limited opportunity for service user involvement [3]. However, research suggests that involving service users in such decisions increases ownership and autonomy which reduces risks to themselves, to others and from others around them.

SDM is widely considered to be an essential part of service delivery in mental health practice across a range of countries. For example, in Scandinavia, service user involvement is enshrined in their first patient law as a right which promotes service users’ integrity, autonomy and participation, this is referred to as Scandinavia’s First Law [16, 17]. SDM can be seen as representative of a broader movement towards person-centred care in mental health services. Psychiatric care has long been criticised for its focus on labels and categories of disorders which are seen as dehumanising and labelling people as deviant without acknowledging subjective experiences of political oppression trauma, ethnicity and culture [18]. Person-centred care embraces the central principle of ‘personhood’, this principle guides the lens through which the individual’s distress and experiences are viewed [19]. A person-centred approach shifts the focus away from diagnoses onto a person’s strengths, values, history, beliefs and considers their identity within the context of their social, cultural and community connections [20].

The increased value placed upon service user’s opinions, preferences and experiential knowledge in SDM is widely endorsed by staff and service users. The SDM agenda has been widely operationalised by the Value Based Practice framework (VBP) which offers a practical model to implement democratic interpersonal approaches to decision making [21]. This proposes true collaboration can only occur when all participants are informed, involved and influential in the decision-making process [22]. However, despite appreciation of SDM, there is growing evidence to suggest that it is not being implemented in clinical practice. Staff report concerns surrounding the competing responsibilities of their role, most notable their professional responsibility for managing risk [22] and challenges around service users’ attitude towards SDM and cognitive capacity, and staff members own willingness and motivation to implement SDM [23]. Clinicians can also face uncomfortable experiences of competing organisational agendas, for example feeling supported to embed SDM even if this means service users are less adherent to treatment recommendations [2]. A qualitative research synthesis exploring attitudes towards SDM reported that service users valued their voice being heard, listened to, and supported to express themselves to professionals [21]. However, service users also reported several challenges to SDM including fear of being judged, lack of trust and feelings of perceived anxiety.

SDM is becoming integral to healthcare delivery in the UK following a supreme court ruling which stated that health professionals must “take reasonable care to ensure that the patient is aware of any material risks involved in any recommended treatment and of any reasonable alternative or variant treatments” [24]. Notably, risk is no longer determined according to the views of a “responsible body of medical men” but by the views of a “reasonable person in the patient’s position”. As such, the National Institute of Clinical Excellence [25] recommends that SDM interventions are offered at multiple stages including before, during or after discussions to ensure people are involved in their care. Although robust models of SDM are highly recommended to address some of the issues highlighted above [25, 26], first-hand accounts of staff and service users experience of SDM in clinical practice within the evidence base are scarce. In situations where SDM is not implemented, service users understandably express a sense of powerlessness and helplessness which can impinge on self-confidence thereby hindering recovery [27]. Strengthening service user involvement through SDM can increase confidence and satisfaction with care [28]. Qualitative research conducted into staff and service user’s experiences of SDM in mental health services suggests that SDM is viewed as an important aspect of service delivery, however there are challenges to embedding such approaches in practice [23, 29, 30]. To date, this has not been synthesised.

## Review aims

The systematic review aimed to understand the attitudes towards and experiences of shared decision-making in mental health services from first-hand perspectives of:

- Service users with personal experience of SDM.
- Staff working in mental health services.

## Methods

### Protocol and registration

The review and protocol were registered on PROSPERO (CRD42023369472). The review was undertaken in line with the Preferred Reported Items and Meta- Analysis (PRISMA) guidelines [31]. Papers were assessed using the Critical Appraisal Skills Programme (CASP) for quality appraisal of qualitative evidence synthesis [32].

### Search strategy

Systematic searches were conducted in November 2022 in APA Psych Info, CINAHL Plus, Medline and Scopus. The following search terms were applied: “Shared decision-making” OR “patient involvement” OR “Shared decision making” OR “SDM” AND “mental health” OR “mental illness” OR “mental disorder” OR “psychiatric illness” AND “mental health staff” OR “mental health nurse*” OR “psychiatric nurse*” OR “mental health social worker*” OR “mental health practitioner*” OR ‘mental health clinician“ OR “service user*” OR “mental health patient*” OR “client*” OR “expert by experience*”. Hand searching was also conducted but yielded no extra papers.

### Inclusion criteria

Articles were included if they were: published in peer review journals between 2010 and 2022; utilised qualitative methods; explicitly reported the experience of or attitudes towards SDM from the perspective of mental health staff from any professional background and/or service users who had accessed adult mental health care (classified as aged 16 or above). Only papers written originally in English or with an easily accessible reliable translation were included.

### Study selection

**Fig. 1:**
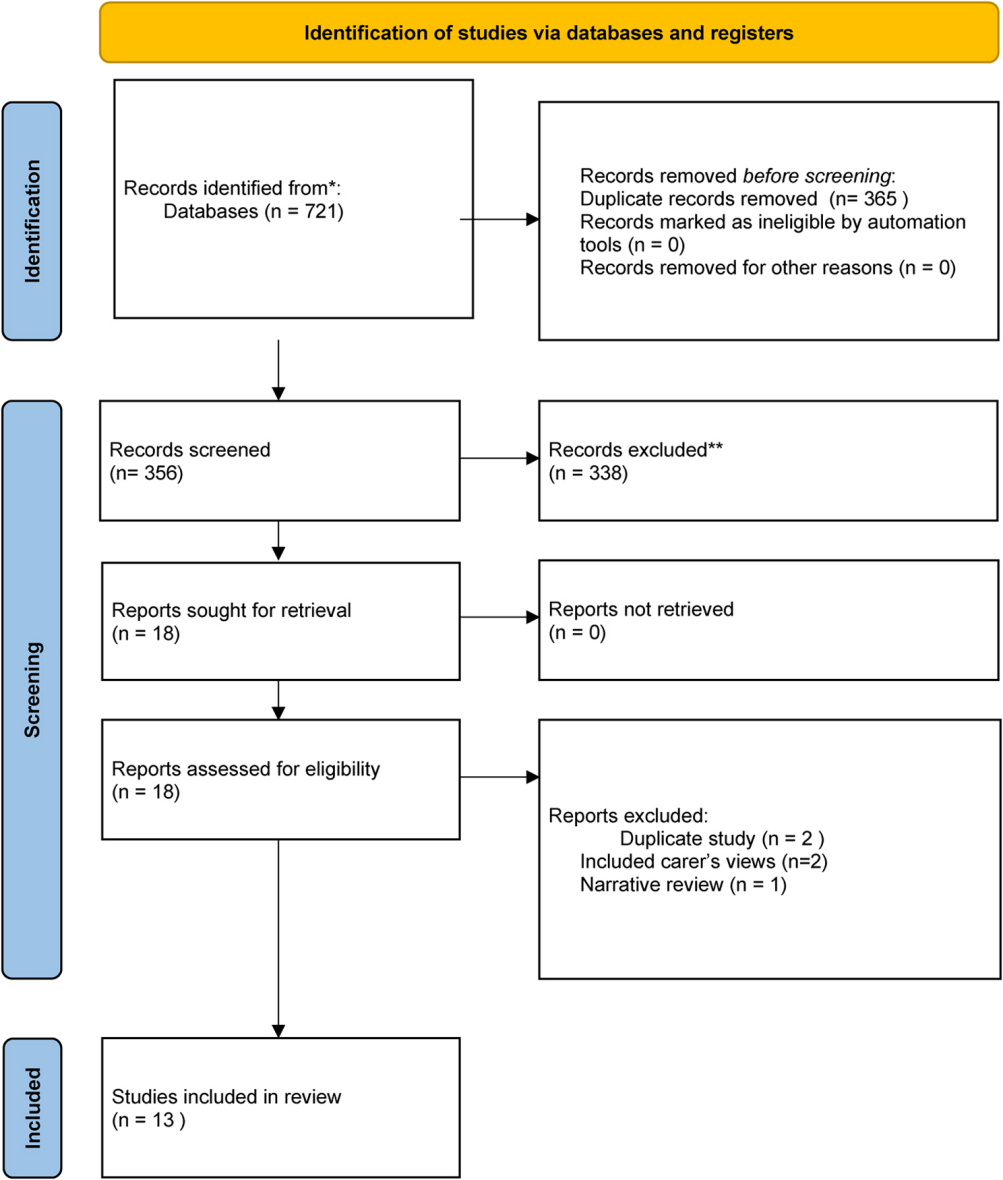
PRISMA diagram for searches of databases

The search yielded 721 articles and after duplicates were removed, 356 remained (see Fig. 1 for PRISMA diagram). Two researchers independently screened abstracts and titles. One researcher (CC) screened all abstracts and titles, and one researcher screened 10% of all selected papers at random (AWG). Both researchers read all remaining papers (*n* = 18) against inclusion/exclusion criteria. Discrepancies were discussed between researchers and any uncertainties resolved by a third researcher.

### Data extraction and synthesis

The following data was extracted: author, year of publication, title, setting, participants, geography, design and conclusions. More detailed data specifically related to the review question, themes regarding participant’s experiences of or attitudes towards shared decision making, outcomes from the research and clinical implications was also extracted. In line with a meta-ethnographic approach, we reinterpreted conceptual data (e.g. themes, concepts or metaphors) created by the primary study whilst considering participant quotes from the primary data [33].

### Quality assessment

Each study was assessed using the Critical Appraisal Skills Programme (CASP) checklist for qualitative synthesis (see Table 1). No papers were excluded from the review based on their quality.

**Table 1:** Characteristics of included studies

Paper	Clear statement of research aims?	Was qualitative methodology appropriate?	Was the research design appropriate?	Was the recruitment strategy appropriate?	Was the data collected in a way to address research question?	Was the relationship between researcher and participant adequately considered?	Were ethical issues considered?	Was the data rigorously analysed?	Was there a clear statement of findings?	How valuable is the research?
Gibson et al. (2020) [34]	Yes	Yes	Yes	Yes	Yes	Yes	Yes	Yes	Yes	Yes
Shepherd et al. (2014) [35]	Yes	Yes	Yes	Yes	Yes	Yes	Yes	Yes	Yes	Yes
Younas et al. (2016) [36]	Yes	Yes	Yes	Yes	Yes	Yes	Yes	Yes	Yes	Can’t tell
Kaminskiy et al. (2021) [37]	Yes	Yes	Can’t tell	Yes	Yes	No	Yes	Yes	Yes	Yes
Becher et al. (2021) [38]	Yes	Can’t tell	Yes	Yes	Yes	Can’t tell	Yes	Yes	Yes	Yes
Klausen et al. (2017) [39]	Yes	Yes	Yes	Yes	Yes	Yes	Yes	Yes	Yes	Yes
Haugom et al. (2022) [40]	Yes	Yes	Yes	Yes	Yes	Yes	Yes	Yes	Yes	Yes
Huang et al. (2021) [41]	Yes	Yes	Yes	Yes	Yes	Yes	Yes	Yes	Yes	Yes
Dahlqvist-Jonsson et al. (2015) [42]	Yes	Yes	Yes	Yes	Yes	Can’t tell	Yes	Yes	Yes	Yes
Knight et al. (2018) [43]	Yes	Yes	Yes	Yes	Yes	Can’t tell	Yes	Yes	Yes	Yes
Woltmann and Whitley (2010) [44]	Yes	Yes	Yes	Yes	Yes	Can’t tell	Can’t tell	Yes	Yes	Can’t tell
Wesseldijk et al. (2021) [45]	Yes	Yes	Yes	Yes	Yes	Yes	Yes	Yes	Yes	Yes
Reed & Jackson (2019) [46]	Yes	Yes	Yes	Yes	Yes	Can’t tell	Yes	Can’t tell	Yes	Can’t tell

## Results

### Data synthesis

Thirteen papers were included in the meta-ethnographic synthesis. This approach enables analysis of the strength and limitations of relationships between themes in the data, in addition to an assessment of the strength of the evidence [47].

### Design and participant characteristics

Please see Table 2 for an overview of studies. The studies were conducted in the UK [34–37], Germany [38], Norway [39, 40], China [41], Sweden [42], Australia [43], USA [44], the Netherlands [45], and New Zealand [46].

**Table 2:** Characteristics of included studies

Author (Year)	Research aim	Location	Setting	Population	Data collection method	Data analysis method
Gibson et al. (2020) [34]	To examine how clients experience SDM within collaborative integrative psychotherapy	UK	University Research Clinic	*N* = 14 Service users = 14	Individual semi structured interviews	Grounded theory
Shepherd et al. (2014) [35]	Sought to explore the attitudes and experiences of SDM in the prescribing of antipsychotic medication	UK	Community mental health teams	*N* = 26 Mental health staff = 26 Consultant Psychiatrists = 26	Individual semi structured interviews	Directed Analysis Method
Younas et al. (2016) [36]	To explore the views and experiences of UK mental health pharmacists regarding the use of SDM in antipsychotic prescribing in people diagnosed with serious mental illness.	UK	Not specified- identified as pharmacists specialising within mental health practice	*N* = 13 Mental health staff = 13 Pharmacists = 13	Individual semi structured interviews	Inductive thematic analysis
Kaminskiy et al. (2021) [37]	Examine views about barriers and enables of SDM.	UK	Community mental health team	*N* = 30 Mental Health Staff = 15 Consultant Psychiatrists = 7 Community Mental Health Nurses = 8 Service users = 15	Individual semi structured interviews	Thematic Analysis
Becher et al. (2021) [38]	To examine barriers and facilitators of SDM with acutely ill inpatients with schizophrenia.	Germany	Inpatient mental health wards	*N* = 32 Mental health staff = 14 Service users- 18	Individual semi structured interviews *Part of a wider RCT in to SDM*	Qualitative content analysis
Klausen et al. (2017) [39]	To contribute to the understanding of shared decision making as an important aspect of user involvement in mental health care from the perspectives of service users	Norway	Community mental health centres	*N* = 25 Service users = 25	Individual semi structured interviews	Thematic analysis
Haugom et al. (2022) [40]	To describe and explore experiences of SDM among patients with psychotic disorders in mental health care.	Norway	Community mental health teams	*N* = 10 Service users = 10	Individual semi structured interviews *Part of a larger study of implementation of evidence-based practice for people with psychosis*	Qualitative Content Analysis
Huang et al. (2021) [41]	To identify mental health professionals’ perceptions of shared decision-making regarding people diagnosed with schizophrenia.	China	Inpatient mental health ward	*N* = 33 Mental health staff = 33 Consultant Psychiatrists = 10 Community Mental Health Nurses = 23	Individual semi structured Interviews and focus groups *Part of a larger study examining perceptions of SDM among people with schizophrenia*,* families and mental health professionals.*	Qualitative descriptive approach
Dahlqvist-Jonsson et al. (2015) [42]	Aim of this study was to explore users’ experiences of participation in decisions in mental health service	Sweden	Not specified- participants recruited based on experience of using mental health services	*N* = 20 Service users = 20	Individual semi structured interviews and focus groups	Constructivist grounded theory
Knight et al. (2018) [43]	To examine how participants reflect on their own experiences of SDM	Australia	Not specified- recruited participants based on lived experience of mental health difficulties	*N* = 29 Service users = 29	Individual narrative interviews	Narrative positioning analysis
Woltmann and Whitley (2010) [44]	Service user decision making preferences and understanding of construction of decisions in community mental health.	America	Community mental health agency	*N* = 16 Service users = 16	Individual semi structured interviews *Part of a larger mixed methods study*	Narrative analysis
Wesseldijk- Elverink et al. (2021) [45]	To examine experiences of service users to enhance SDM	Netherlands	Inpatient mental health ward	*N* = 13 Mental health staff = 7 Service users = 6	Individual semi structured interviews	Reflective lifeworld approach
Reed and Jaxson (2019) [46]	To investigate the experiences of qualified mental health practitioners in using a shared decision-making approach	New Zealand	Mental health and addictions agency	*N* = 4 Mental health staff = 4	Individual semi structured interviews	Thematic analysis

Most participants were recruited from community outpatient mental health settings (*n* = 200), with some data from acute inpatient environments (*n* = 73). Across all papers, a total of 153 service user’s views were sought, alongside 112 staff members. From the 13 studies, 6 included the views of only service users, 4 included the views of only staff and the remaining 3 included the views of both staff and service users. Staff including psychiatrists, pharmacists, and mental health nurses were recruited. Unfortunately, staff member profession was sometimes not provided and is therefore classified as ‘other’ (*n* = 38). Papers that recruited service users were limited by only examining the views of those formally diagnosed with psychosis. Other demographics such as participant gender, age and ethnicity were inconsistently collected and reported, and therefore could not be synthesised.

Twelve papers collected data using semi-structured interviews. Two papers used focus groups alongside interviews [41, 42], and one used narrative interviews [43]. Four studies were part of larger mixed method studies [38, 40, 41, 44]. Data analysis methods varied between papers, including thematic analysis [36, 37, 39, 46], content analysis [38, 40], grounded theory [34, 42], descriptive approaches [41], narrative analysis [43, 44], a directed analysis [35] and a reflective lifeworld approach [45].

Two studies explored experiences of and attitudes towards SDM in medication prescribing in mental health care from the perspective of staff [35, 36]. Two papers explored barriers and facilitators of SDM from the perspectives of staff and service users on an inpatient ward [38] and in a community team [37]. Remaining papers explored general experiences of shared decision making in mental health services (*n* = 9). Four explored service user’s experiences of shared decision making whilst under the care of community mental health teams [34, 39, 40, 44]. One study looked at service users and staff experiences of SDM on an inpatient ward [45] and a further two only explored service user’s views without specifying the setting [42, 43]. Two papers explored only staff experiences of SDM, one on an inpatient ward [41] and one in a community team [46].

*A meta-ethnographic approach was used* [33, 47]. *This systematic approach was chosen*,* as it allowed us to synthesise data from each study to provide insights that could improve SDM-based healthcare service delivery* [33]. *The seven steps of meta-ethnography were followed enabling us to define the scope of our review (Phase 1)*,* locate*,* screen and quality assess studies (Phase 2) before reading each study (Phase 3). We then moved to determining how the studies are related (Phase 4) by identifying and drawing comparisons between key concepts presented in each study. Studies were then translated into one another*,* by establishing similarities and differences between concepts identified in each paper (Phase 5)*,* and these translations synthesised to develop a framework (Phase 6). Finally*,* we wrote up our synthesis and identified clinical implications (Phase 7)* [33, 47].

Data was synthesised to develop first and second order constructs. A list of key concepts from each paper were then developed. We also labelled whether the study had included service users, mental health staff or both groups. Each concept was compared to all other papers to check for presence or absence of commonality [33]. This process was followed until a synthesis of first and second order interpretations were complete. Concepts were then synthesised into clusters to produce a reciprocal translation synthesis (see Table 3).

**Table 3:** Translations table for meta-ethnography

Descriptor (groups of similar concepts clustered together/ broad thematic headings	First order data (participant quotes/ primary data from the studies)	Second order (themes developed by primary authors)	Third order (higher order concepts)
**Service user involvement**	“We use different life domains on which the patient chooses his actions. We discuss it together, planning small steps to reach progress toward larger ends. Who am I to tell a patient that a goal is not achievable…I believe it is a (learning) process, for both of us.” [45] “It is really client directed, it’s really what they want to do rather than what I want them to do” [46] “You ought to think about openness and honesty when working in mental health service. They decided the treatment … and the user; it was like you could say your opinion, and you sort of … I felt, I felt it was a good treatment. I was a part of making the decisions. I LIKED THAT A LOT” [39] “But I just think if I’d been given that information and going through it yourself and having time to discuss it, you’re going to understand. I just think you’d feel like you had more control and, you know, that might reduce stigma, as well as you feeling you can take control of what’s going on.” [37]	Mutual understanding [45] Knowing the client [46] Shared decision making in- admission, individualised treatment, different treatment contexts [39] Consumer perspectives on decision making in case management [44] Inward expert- self as expert in own presentation [43] Self – aware observer- greater confidence in own decisions despite expert advice [43] Being involved in shared decision making-being omitted, controlled or considered the underdog [42] Willingness to engage patients in shared decision making [41] Participation as desirable and achievable [40] Experiencing decisions as shared [34] Facilitators and barriers towards shared decision making-patient related factors [38] Enacting shared decision making in service user/ provider interactions [37]	***The role of service user ownership***
**Diagnosis impacting mental capacity**	“I didn’t find it that onerous. I, I think that the community treatment order [worked] well for me, all it was, was just getting an injection every two weeks. . I didn’t fight it because I didn’t want to go back to the voices.” [43] “I think the biggest barrier with her is her complete and utter lack of suffering, there just isn’t any. She also doesn’t realize that she creates suffering in others, in her environment. She simply isn’t accessible to any rational discussion.” [38] “They might be acutely unwell…they might not be in a position to make a decision they might be forced to have treatment against their wishes so in that scenario you’re not going to be able to provide them with SDM.” [36] “I don’t know. I think sort of being asked was quite daunting … But you go from sort of quite daunting like “I want support but I don’t know what support.” And then like, being given that small amount of support like calms you down a bit because you’re being shown what support you’re getting.” [34] **“**Some patients are just too unwell to make that kind of decision, they can have no capacity at all to make that kind of decision at the time of admission, in which case we just have to go with what we feel is advisable at that time.” [35]	Conflicts with decision making authority [45] Outward entrustor- looking to medical expertise for guidance [43] Barriers to implementation [36] Perceiving shared decision making as unachievable [41] Varying degrees of involvement [40] Deciding on treatment options [35] Daunting for clients to be asked to take part in difficult decisions [34] Treatment decisions and stages of participation- no participation to beyond participation [38]	***The role of fluctuating capacity***
**Trust and collaboration between service user and staff**	“I think the main thing is to be as honest as possible….the honesty and the trust I think as well, and you know you kind of build up a relationship with somebody and you get to trust them.” [37] “I may be the expert, but I don’t know how to apply that knowledge, [Psychotherapist] does. So, it makes sense to just kind of let [Psychotherapist] suggest stuff and me occasionally suggest stuff when I’ve got a better understanding of what we’re talking about.” [34] “It’s about getting… Getting a person where there’s good chemistry. Someone you can trust so you can tell them how you’re feeling. You have to feel that you, like, you have a kind of, you know, trust, that you… I mean that you can trust someone and you feel you can talk to them. Because you don’t get on in the same way with everybody. When you want to… so it’s good.” [40] “It is important to build rapport with your clients to create a level of respect and engagement… this will create an environment that a client is ready and willing to set, and work towards goals.” [46]	Therapeutic relationship as an enabler of shared decision making [37] Facilitators and barriers towards shared decision making- clinician related factors [38] Therapists supporting clients to become more active in decision making process [34] Clients felt recognised as an individual and accommodated by psychotherapist [34] Deliberation [35] Shared decision making requires a trusting relationship [40] Benefits of shared decision making [36] Participants views on case manager role in decision making [44] User/professional relationships [39] Therapeutic relationships [46] Bridging the therapeutic gap [45]	***The importance of therapeutic alliance***
**Clinicians impact on SDM**	“It is often difficult to find time to plan and prepare a session with a client to ensure clear and consistent treatment.” [46] “We used a directive approach and convinced him he is somewhat lazy. Before, we ruled out other things too, such as fear or suffering from negative symptoms… You have to be careful not to oversee these factors, it opens a broader perspective. It allows us to meet the person instead of taking things over because he is obviously not doing it by himself… and then we can make a strong case against the patient… How are you able to life on your own if you are even not able to come out of bed with help from us?” [45] “Our culture has advocated obedience. . for patients, experts and professors are authorities who should be respected…treatment decisions are up to doctors, which is a tradition…” [41] “Some of the clinical teams I’m in are very collaborative and very collegiate… I’ve also worked in teams where there’s very little conversation apart from between nurses and doctors, me as the pharmacist has to almost fight to say something” [36].	Structural challenges to achieving shared decision making in practice [37] Barriers and facilitators to shared decision making- clinician and settings factors [38] Both parties presenting and recognising expert knowledge [34] Information sharing [35] Shared decision making requires a trusting relationship [40] Role of mental health pharmacists [36] Perceiving shared decision making as unachievable [41] Conflicts with decision making authority [45] Awareness of the practitioner [46]	***Changing clinician’s behaviour and attitudes***

### Overview of higher order concepts

All studies broadly supported the concept of shared decision making (SDM) in adult mental health services, while acknowledging and reflecting on challenges to embedding such interventions in clinical practice. This is reflected in the scarcity of the literature available evaluating the quality and effectiveness of SDM, despite being recommended and advocated as best practice [25, 26]. The author identified four main concepts which were evident in each study (see Table 3). The relationship between the concepts as described in each study were considered, examined, and interpreted by the researchers. These are outlined below:

### The role of service user ownership

All studies expressed positive views on service user ownership over decisions in their clinical care, particularly about medication. However, in most studies, service users reported they were not involved in such decisions. Service users reported that they were not given sufficient information from healthcare providers to fully engage in SDM despite expressing a preference to be included in decisions [2]. Strategies that promote ownership and self-determination were reported to engage service users in becoming active participants in their care. For example, asking service users what getting better means for them would elicit some of the person’s values and perspectives, which may help to formulate a shared care plan that considers what is valuable to the service user whilst balancing the clinician’s perspective of the person’s needs [48].

Clinicians felt that openness and active communication were important factors for service users [35, 36, 41]. Personality traits, including empathy, assertiveness, and willingness to compromise and cooperate, were noted by clinicians as possible influences on the willingness of service users to engage in communication [34, 40]. Conversely, lack of motivation and reluctance to be open to clinician’s suggestions were potential barriers and were seen as static properties of the individual. Several papers [34–37, 40, 41] recommended that clinicians should improve service user’s knowledge by sharing information more readily, as lack of information was associated with feelings of helplessness and loss of control. In all studies, the target of the suggested intervention or behaviour change was the service user, rather than individual staff members, or the staff team. Some papers suggested clinicians should consider the importance and impact of power dynamics existing within the mental health system and seek to understand feelings of powerlessness amongst service users [37, 45].

Service users’ aspirations regarding SDM varied. In some accounts, service users described being ‘experts’ in terms of their knowledge about their mental health and others described high feelings of dependence on clinicians [37, 40, 43]. There was also fluidity between their positions. Service users and staff reflected that during times of crisis, service users may feel more comfortable with staff taking more ownership in decision making. It seems clear from the research that this should be an ongoing discussion between clinician and service user with reasonable adjustments made to facilitate engagement where possible and all decisions should be made in the service user’s best interests, and the papers suggest the same. However, few of the papers offer discussion on the implications of the structures and cultures of mental health care, and how feelings of dependence in times of need may highlight a shift in power dynamics and change service user’s preferences in SDM. For example, when a service user is experiencing a mental health crisis, they may prefer clinicians to have more influence over decisions they consider to be in their best interests [36, 43].

One paper examined service user experiences of SDM in psychotherapy settings [34], where service users experienced shared decisions, felt comfortable in decision making and felt recognised as an expert. This suggests differences in service user ownership between settings [2]. For example, decisions within a psychotherapeutic framework can be relatively complex and based upon client’s difficulties with psychotherapist interpretation which requires joint exploration within a therapeutic alliance. By contrast, interactions with other mental health services may require fewer abstract decisions where a service user presents a series of symptoms to receive a diagnosis and subsequent treatment [34]. A key factor in several papers was service user ‘readiness’ to engage in treatment [39–41]. As psychotherapy requires, and depends upon, client collaboration this may be the reason why people felt more able to engage in SDM.

One study interviewed service users and staff following a SDM training intervention for staff and service users [38], finding that SDM had been embedded on the inpatient ward following this intervention. Training in SDM may be a suitable intervention for both staff and service users in adult mental health care.

### The influence of fluctuating capacity

Impaired decisional capacity due to symptoms of mental illness was a barrier to embedding SDM within healthcare settings by both staff and service users [35, 36, 39, 45]. This included perceived inability of service users to participate, due to a lack of ability to be reflective or communicate effectively with staff members. Clinicians often highlighted limitations to service user engagement due to their symptoms [37, 41, 43]. This was particularly highlighted in studies conducted in inpatient settings. For example, mental health nurses in inpatient wards stated that service users were ‘too unwell’ to have active involvement in SDM. Some clinicians reported trying to incorporate collaboration by using basic actions such as going for a walk or having conversations with a service user, rather than seeing this as a more formal process [39]. Service users who had been diagnosed with serious mental illnesses expressed the most distrust and feelings of powerlessness in relation to the healthcare system. However, these participants still wished to be involved in decisions about their care.

Some staff members in the reviewed studies suggested that the service users they work with are too ‘high risk’ or ‘vulnerable’ to be considered appropriate for SDM. In such cases, it was recommended that SDM approaches could be adapted [35, 38, 42]. It was argued that when people are most unwell, they are at the highest risk of feeling disempowered, therefore encouraging a service user to make even basic decisions is a way to provide a sense of control and increase motivation towards recovery. Participants reflected that this requires a high level of skill from the clinician in balancing safety and risk. The included papers make recommendations that mental capacity or ‘insight’ is not static and requires a dynamic dialogical approach, within which the goal should always be SDM. Service users had experienced attitudes which conveyed that ‘professionals know best’ and their views were not welcomed within a decision-making context [41, 43, 44].

### The importance of therapeutic alliance

High quality and meaningful therapeutic relationships were highlighted in all included studies as a facilitator of SDM. Service users expressed a strong desire for clinicians to provide more than medical treatments and provide more therapeutic spaces to be empathic and insightful in supporting them to overcome their distress. The papers suggest the therapeutic process is based on trust, collaboration and sharing mutual aims and goals [34, 36–38, 45]. Whilst good relationships facilitate SDM, it is suggested that the therapeutic alliance is strengthened by good quality SDM approaches. Continuity of staff and service user relationships was viewed as one of the most desired components of care, as this encouraged people to share their stories openly without fear of negative repercussions [39, 42, 46]. When relationships appeared to be trustworthy and caring, service users were more open and understanding towards clinician’s decisions even when there were disagreements. Service users considered care that was compassionate and empathic to be instrumental in regaining a sense of independence and increased their ability to be more actively involved in SDM.

Clinicians also emphasised the importance of mutual understanding to increase collaboration and goal setting. Staff reported that the more beneficial outcomes they observed from the choices made by service users, the more they were inclined to respect the autonomy of the service user [37, 38, 45]. It was found that in complex inpatient settings, even basic principles of SDM can be used to enhance therapeutic collaboration. Furthermore, service users felt it was important for SDM to be incorporated in all mental health settings, at every point in their recovery journey.

Medication was the most dominant discourse regarding treatment in most of the included studies and two studies focused solely upon SDM in prescribing of antipsychotic medication from the viewpoint of pharmacists and psychiatrists [35, 36]. Both studies discussed the importance of information sharing regarding medication side effects and health implications for service users to make an informed choice. Pharmacists felt more able to participate in SDM as they were external to wider healthcare teams, but felt their role in SDM was under-utilised. Service users understandably have less adherence to medication when they feel they have not been adequately involved in decision making to take them.

One study described the importance of considering cultural features to make SDM culturally feasible and acceptable, particularly in regard to considering therapeutic alliances. Most studies into SDM have been conducted in western cultures and more research is needed to fully understand cultural issues that may present barriers to SDM [41]. SDM approaches are becoming increasingly common and international recognition of the importance of SDM is considered to reflect the advancements in mental health care through service user involvement movements across the world.

### Changing clinicians’ behaviour and attitudes

Some clinicians expressed scepticism towards SDM approaches, particularly staff working in inpatient settings. It was found that some staff placed more value on safety, adherence and medication and felt this was conflicted with the values of SDM. Some staff also expressed a lack of trust in service user’s abilities to be actively involved in their care and make wise decisions. Clinicians often reported being in dilemmas with competing demands between duty of care and promoting independence for service users. They felt challenged in trying to ensure that both were being always respected. In some cases, limited time and resources contributed to an inability to reflect on this, despite there being a wish to have this time to reflect. Staff participants reported that funding issues and organisational changes made it difficult to provide person centred care more broadly, which impacted their ability to engage in SDM with service users [37, 38, 46]. Person centred care refers to an approach which is responsive to and individual’s personal circumstances, values, needs and preferences. Due to restrictive practices, service users found they were omitted from decision making which they reported increased their feelings of anger and hopelessness with care providers. Conversely, service users within the least restrictive setting, the psychotherapy department, talked about consistency in experiences of positive attitudes towards SDM from clinicians.

The reviewed papers suggest clinicians should be sensitive to the needs and wants of the individual for true SDM to be achieved [39, 44, 45]. Service users emphasised the need to be relational in SDM with equal contributions from clinician and service user, wishing to be seen as an equal partner in the relationship. However, most service users acknowledged a desire to delegate decisions to professionals when they feel they need to, which may shift the balance of equality. It is key that clinicians reflect upon their own values and belief systems to consider the impact this has on their ability to share decision making responsibilities with service users. Positive encounters in these settings enhance service user’s sense of agency, which in turn supports personal recovery. Recognition that service user’s needs, ability to engage with, and desire to be involved in, shared decision making may fluctuate over time requires an empathic, sensitive clinician, who knows the service user well and is able to understand their viewpoints [37].

## Discussion

Despite recommendations for SDM to be implemented in clinical practice, there are a lack of first-hand accounts of this. This meta-ethnographic review provided an in-depth higher order interpretation of the existing literature. Only thirteen qualitative studies exploring experiences of and/or attitudes towards shared decision making in adult mental health care settings, from the perspectives of mental health staff and/or service users were identified.

We found that mental health staff and service users see SDM as good practice in adult mental health care. This is in line with UK policy guidance and recommendations for SDM by NICE [25], NHS England [26], the Mental Health and Act (1983) [8] and the Mental Capacity Act (2005) [49]. Similar policies and recommendations exist in other countries, although their presence is inconsistent in the papers and should be systematically explored. The included studies highlight several complexities and challenges to embedding SDM approaches in clinical practice. Notably, concerns about service user’s capacity to engage, staff attitudes towards SDM and limited time and resources to invest in these approaches [35, 36].

The first theme of the review centres around service user ownership of their involvement in SDM. All the included studies highlighted the importance of service user ownership in SDM, a view held by both staff and service users. The participants in one study emphasised the importance to service users of having a sense of control over their lives [42], making the important link that as mental health care is a significant aspect of people’s lives, most people would reasonably expect decision making to be a mutual process. In one paper, staff, psychiatrist and nurses all strived towards increased ownership, associating it with working towards self-management, and a process of involving service users in decisions [35]. Feelings of control for service users in this study was increased through detailed information about the options service users had. Importantly, the papers also document the feelings of helplessness and lacking control resulting from not feeling involved in decisions such as changes in medication. While psychiatrists and nurses in this study valued service user ownership, the presentation of information seems to be the key mechanism by which ownership is reduced. This is often subtle, such as psychiatrists redirecting service users to leaflets about adverse medication effects rather than engaging in a wider discussion. This is an important point and is evidence of how well-meaning staff may unintentionally undermine shared ownership and SDM. There are likely underlying assumptions being acted upon here, the deconstruction of which could aid the development of interventions based at increasing SDM. Service user movements have significantly changed the role of the ‘patient’ in their own care [50]. Policymakers have responded to this by introducing frameworks which emphasises person-centred care based on individual’s circumstances and choices. Therefore, achieving SDM involves a full recognition of the person’s expertise developed through their own experience of their mental health problems [21]. It is suggested that service users who are active participants in their care have overall better engagement which leads to more positive outcomes [2, 51]. Therefore, increasing service user ownership through SDM could lead to improved satisfaction with care, although this remains to be explored.

The second theme highlights the importance of fluctuating mental capacity of the service user and the impact this could have on SDM. This was particularly evident when staff considered the service user to have a serious mental health difficulty such as a diagnosis of ‘psychosis’ [39, 42], or had been admitted to an inpatient service due to mental health crisis [35]. It was suggested the characteristics of such mental health presentations would have a more significant impact on the service user’s ability to engage in SDM. In fact, the construct of insight can simply become the frame through which professionals view, and therefore challenge, the service user’s competence to participate based on the increased need for information and guidance when experiencing a crisis [37]. Symptomology affecting decisional capacity and communication with people experiencing psychosis was the barrier to SDM most cited by staff the Becher and colleagues study [38].

Service users in the Kaminskiy and colleagues study [37] however don’t refer directly to insight, but speak in more functional terms, describing poor concentration and memory problems – not excluding SDM but highlighting the increased need to support the person with information and guidance. Principle 2 of The Mental Capacity Act (2005) [49] requires practitioners to help a person to make their own decisions before deciding they’re unable to decide. This means that practitioners should take an active role in supporting the service user with their memory or communication to reduce their distress and help them to understand and weigh up information relevant to their decision [25]. Although this is recommended, research suggests that SDM is not implemented routinely in clinical practice [2]. NICE does offer clear guidance on how to implement SDM in mental health settings which also incorporates the importance of SDM at a cultural, organisation and strategic level [25].

Some studies have highlighted that service users would prefer staff to lead on clinical decisions when they feel their capacity is impaired by their mental health difficulty [52]. Service users emphasised the importance of building trusting relationships with staff to allow them to make decisions in their best interest [42]. Mental capacity is part of the wider concept of decisional capacity which itself has several elements. Other contributing factors could include confidence in one’s own decision-making ability, apprehension of the consequences of decisions, or lacking trust in the relationships. Haugom [40] suggests that shared decision making increases this decisional capacity. Though the mechanism isn’t clearly described, this review would suggest the influences are a combination of improvements made within the organisational systems while enhancing individual elements such as personal confidence. This suggests no clear distinction should be made between the organisation and the individual here, as each always exerts an influence on the other.

An important theme emerging from the review is that of the therapeutic alliance, and its contribution to shared decision making. Definitions vary, but most include a sense of warmth and openness, emotional bond, and shared expectations of the goals and tasks of therapy [13]. A key element, and perhaps linked to that of shared expectations, is mutual collaboration [14]. There is overlap both in the language and practical application of therapeutic alliance and shared decision making. SDM is an act of mutual collaboration, which in turn is described as a key element of, or contributing factor to therapeutic alliance. While therapeutic alliance could be considered the quality of the relationship, and shared decision making an action taking place within that relationship, the literature seems to suggest the two are inextricably linked, with improvements in one contributing to improvements in the other [37, 45]. Further work is required to provide clear definitions of how the two constructs may interact. Therapeutic alliance is considered an integral part of most if not all established psychotherapy models, which operationalise within the model collaboration and development of a trusting relationship. Much of the research on SDM focuses on medical settings, and further research could look at the possibility that psychotherapy models are operationalising not just alliance but the act of shared decision making albeit through different language.

However, there are several factors which may impact the ability for staff and service users to engage in SDM. Decisions in mental health settings are complex to navigate for both staff and service users therefore it takes considerable time to develop a trusting relationship and learn what is important to the service user [53]. Organisational and cultural constraints on mental health staff can mean they do not feel confident in involving service users in decisions about their care. Potential barriers cited in the research include limited time and resources to make reasonable adjustments, inadequate training in suicide prevention, fears about an adverse reaction from those considered to be a risk to others and fear of culpability and litigation [54].

Lastly, the review outlined the importance of considering organisational influences on the implementation of SDM. It is suggested that paternalistic practice and disciplinary forms of power are prevalent within the mental health system, and this presents significant challenges to achieving SDM in practice [23, 55]. De las Cuevas and colleagues discuss this phenomenon in Latin America in particular, additionally highlighting that such views are more prevalent in older generations, perhaps hinting that changes are already taking place [56]. Attitudes should be viewed within a culture, so it is unsurprising that overall sentiment towards SDM appears to be ambivalent, with only situational or topic dependent endorsement [57]. Indeed, paternalism and mutual collaboration seem at first glance conflicting, perhaps this ambivalence is a hint at this conflict in the individual, as influenced by organisational structures. Service user reports also highlight these organisational issues [39, 44]. People often report themes of helplessness, being omitted from decisions, and being controlled, when describing their experiences of SDM in mental health care [37, 42]. People also reported the perception that their views were less valuable than those of the practitioner [42, 58]. This perhaps mirrors some of the above noted staff perceptions (again as influenced by the cultures) that service users have a reduced capacity to make decisions, or indeed that there are risks in increasing their ability to do so, hence feeling the need for paternalistic control over decision making. There was also no reflection on the potential influences such views could have if pervasive through a certain setting. Clinicians placed emphasis on considering service users fluctuating capacity to engage in SDM, however were less reflective on their own individual biases towards SDM or organisational barriers [48].

Current literature suggests that on the whole service users, across cultures would prefer to engage in SDM rather than being engaged with services which paternalistically act on their behalf [41]. However, there are several cultural factors that should be considered for SDM to be embedded effectively. For example, in China there are deep rooted cultural values which emphasise the importance of respecting authority and maintaining harmonious relationships [59]. Therefore, people using mental health services in China may perceive some of the principles of SDM as a ‘challenge’ to the authority of the mental health system and be more apprehensive to engage.

Family involvement in SDM is also considered to be more important in more family-orientated cultures. For example, in countries such as China, Korea and Ethiopia treatment decisions are very rarely made without input from the service user’s family [29]. Latin American service users also expressed a higher preference for family to be involved in their care [60]. This highlights the importance of involving families and significant others in decision making across cultures.

Overall, the papers suggest successful implementation of SDM approaches in mental health care will contribute to higher quality, more effective care, particularly when considering risk management in mental health care. Whittington and colleagues [9] suggest that collaborative approaches to understanding risk in mental health services, lead to more positive experiences for the service user, increasing engagement with services which leads to more effective risk management strategies and decreases the likelihood of high-risk behaviours. Conversely, defensive practices that do not subscribe to an SDM approach increase the likelihood of high-risk events occurring due to lack of engagement from service users.

## Clinical implications

Staff and service users alike voice positive regard towards SDM, and its importance in developing a strong therapeutic alliance, as well as increasing the quality and effectiveness of mental health care delivery. However, very few experiences of its application in adult mental health care have been reported to date. A combination of practical barriers and cultural attitudes have been identified. Interventions to increase SDM will likely need to address both staff and service user experiences in parallel, as the services and systems that currently don’t allow for opportunities for SDM both influence and are influenced by these embedded attitudes. Therefore, it is not as simple as creating time for SDM, but may include educational interventions such as staff training, or systemic changes such as processes that make use of those decisions. This will avoid staff perceiving initiatives as frustrating and unnecessary [53].

Co-developed, and co-delivered, staff training seems necessary if skills development and attitudinal changes are to be achieved in staff groups delivering care, especially in challenging and pressured environments such as acute inpatient units. This training should aim to share the first-hand experience of care in these environments, both of being engaged in and being denied the opportunity to engage in SDM. Co-developed and delivered training has been shown to increase empathy and attitudes amongst staff [23]. This also gives an opportunity for staff to consider perceived individual behaviours and barriers against responses to environmental factors. Hearing first-hand that an inpatient environment might be experienced as restrictive and frightening, and oneself vulnerable in that environment, especially whilst experiencing psychotic phenomena, may challenge the idea that people on the ward are simply withdrawn or not willing to engage. Staff may come to understand that in this context, the experienced behaviour, rather than symptomology, could be fear and mistrust that importantly could be overcome through strategies that emphasise empathy, warmth, and development of a trusting relationship. The higher idea to be embedded is that a person’s ability to engage in SDM is not static but can be developed despite their current mental health difficulties, through changes to the environment and how we work - not just what we do, but how we do it, and the space we do it in.

Consideration should be given to the issue of the mental capacity to engage in SDM in the context of service user’s current difficulties, given its frequent indication by staff in the literature as a reason for not engaging in SDM [35, 36]. Lacking or fluctuating capacity will certainly be an issue in environments like inpatient wards, however, training and processes should emphasise that it does not preclude joint decision making. For those who are considered to lack capacity interventions such as advanced statements, the involvement of family and carers (ideally pre-agreed), and least restrictive human-rights based approaches could be introduced and made common practice. Processes should necessitate careful consideration of mental capacity and the implementation of associated legal frameworks.

At the service level, consideration needs to be given to how SDM takes place, the spaces it takes place in, how much time is needed, and who leads this. Third parties could be helpful in promoting these practices, such as psychologists tasked with facilitating such approaches; core staff could take a lead supported by such external agents to ensure shift in general sentiment and value of SDM. An audit of current levels of SDM within services could begin implementation.

Engagement in SDM should also be considered within a framework in the system in which it is used. There are key decision points in services, often involving concerns about risk, for example leave, discharge, or medication. Embedding SDM frameworks around these decision points could ensure SDM takes place, is documented appropriately, and most importantly is acted on. This could involve semi-standardised approaches that ensure wishes are documented alongside discussions of concerns and compromises that necessarily need to be made. An example might be the careful consideration necessary for a person requesting leave though there is a considerable risk of the person harming themselves. It would be important here to document the wishes of the person, the importance of this to the person, and the importance of supporting the person from a human-rights perspective. The concerns of the person taking leave would need to be clearly articulated not in documentation but to the person, and it would help to document the discussion, any negotiations, compromises and final decisions.

While these approaches are aimed at staff, many service users reported lacking confidence to engage in SDM. Interventions could be targeted at the individual level, coaching the person through the processes involved. Emphasis should be given to the ability to *develop* the person’s capacity for SDM, and staff supported to develop their skills.

Many mental health systems have traditionally operated on authoritarian and paternalistic principles: namely that we care for people by taking control and making decisions because the person has reduced capacity to. For SDM to become common practice, these ideas must be challenged. The above strategies and interventions could aid this, moving towards a culture of sitting alongside the person to support their journey.

Finally, any intervention implemented must be researched for its outcomes, with process in place for adjusting ineffective elements. As Knight and colleagues [43] highlighted, there is limited research on the effectiveness of interventions designed to improve SDM. It is important to innovate to improve the quality of care, but where strategies have little supporting evidence, we must implement them systematically to ensure they are successfully achieving their aims.

## Strengths and limitations

This is the first systematic review to look at *both* staff and service user’s experiences of, and/or attitudes towards, SDM in adult mental health care. This allowed an exploration of similarities and differences between the narratives around staff and service users perspectives on SDM. We hope this will increase understanding and contribute towards developing robust SDM interventions which are more effective in clinical practice. The review was conducted within PRISMA guidelines, and three reviewers screened papers. The CASP quality assessment tool was used to assess methodological rigour and quality. Furthermore, the review provides evidence that SDM improves service user’s and staff experiences of mental health care. The papers included in this review are from a variety of countries which gives some insight in to how SDM is being incorporated within different mental health systems across the world. However, most included countries were westernised, so some biases will exist. Therefore, attempts to generalise this work should be done so cautiously, and further reviews examining staff and service user’s experiences of and/or attitudes towards SDM are warranted.

Despite the positive indications from this review findings should be treated cautiously. Comparison between studies was difficult due to different data collection and analysis methods. It is also notable that four studies were part of larger mixed methods studies, with the quantitative data published separately. Studies were also conducted with staff groups from different disciplines across different clinical settings making comparison difficult.

Further research should be conducted to understand why SDM is not commonly occurring in practice despite being advocated for in policy and literature. The review highlights the need to consider wider implementation of SDM approaches to ensure person-centred recovery practices are routinely embedded in adult mental health services. Structured tools for implementing SDM which incorporate the quality of the relationship between service users should be established.

## Conclusion

In conclusion, this review identified that service users and staff support the use of SDM in clinical practice. Despite the policy support for SDM, it has not been widely implemented in mental health care yet. However, a wide range of barriers to implementation were identified in the review, such as scarce time and resources, lack of understanding of SDM, lack of SDM models and training and service user capacity, which could be addressed through a range of clinical interventions and practices. This review presents unique perspectives of SDM from mental health staff and service users which can provide useful insights into the strengths and limitations of current SDM practices. These insights could be used to develop and implement SDM models in adult mental health care settings.

## Data Availability

No datasets were generated or analysed during the current study.
